# Promotion of lung adenocarcinoma following inhalation exposure to multi-walled carbon nanotubes

**DOI:** 10.1186/1743-8977-11-3

**Published:** 2014-01-09

**Authors:** Linda M Sargent, Dale W Porter, Lauren M Staska, Ann F Hubbs, David T Lowry, Lori Battelli, Katelyn J Siegrist, Michael L Kashon, Robert R Mercer, Alison K Bauer, Bean T Chen, Jeffrey L Salisbury, David Frazer, Walter McKinney, Michael Andrew, Shuji Tsuruoka, Morinobu Endo, Kara L Fluharty, Vince Castranova, Steven H Reynolds

**Affiliations:** 1 National Institute for Occupational Safety and Health, 1095 Willowdale Road, Mailstop L-3014, Morgantown, WV 26505, USA; 2 Integrated Laboratory Systems, Durham, NC, 27713, USA; 3 University of Colorado, Aurora, CO, 80045, USA; 4 Mayo Clinic, Rochester, MN 55905, USA; 5 Shinshu University, Nagano, 380-8533, Japan

## Abstract

**Background:**

Engineered carbon nanotubes are currently used in many consumer and industrial products such as paints, sunscreens, cosmetics, toiletries, electronic processes and industrial lubricants. Carbon nanotubes are among the more widely used nanoparticles and come in two major commercial forms, single-walled carbon nanotubes (SWCNT) and the more rigid, multi-walled carbon nanotubes (MWCNT). The low density and small size of these particles makes respiratory exposures likely. Many of the potential health hazards have not been investigated, including their potential for carcinogenicity. We, therefore, utilized a two stage initiation/promotion protocol to determine whether inhaled MWCNT act as a complete carcinogen and/or promote the growth of cells with existing DNA damage. Six week old, male, B6C3F1 mice received a single intraperitoneal (ip) injection of either the initiator methylcholanthrene(MCA, 10 μg/g BW, i.p.), or vehicle (corn oil). One week after i.p. injections, mice were exposed by inhalation to MWCNT (5 mg/m^3^, 5 hours/day, 5 days/week) or filtered air (controls) for a total of 15 days. At 17 months post-exposure, mice were euthanized and examined for lung tumor formation.

**Results:**

Twenty-three percent of the filtered air controls, 26.5% of the MWCNT-exposed, and 51.9% of the MCA-exposed mice, had lung bronchiolo-alveolar adenomas and lung adenocarcinomas. The average number of tumors per mouse was 0.25, 0.81 and 0.38 respectively. By contrast, 90.5% of the mice which received MCA followed by MWCNT had bronchiolo-alveolar adenomas and adenocarcinomas with an average of 2.9 tumors per mouse 17months after exposure. Indeed, 62% of the mice exposed to MCA followed by MWCNT had bronchiolo-alveolar adenocarcinomas compared to 13% of the mice that received filtered air, 22% of the MCA-exposed, or 14% of the MWCNT-exposed. Mice with early morbidity resulting in euthanasia had the highest rate of metastatic disease. Three mice exposed to both MCA and MWCNT that were euthanized early had lung adenocarcinoma with evidence of metastasis (5.5%). Five mice (9%) exposed to MCA and MWCNT and 1 (1.6%) exposed to MCA developed serosal tumors morphologically consistent with sarcomatous mesotheliomas, whereas mice administered MWCNT or air alone did not develop similar neoplasms.

**Conclusions:**

These data demonstrate that some MWCNT exposures promote the growth and neoplastic progression of initiated lung cells in B6C3F1 mice. In this study, the mouse MWCNT lung burden of 31.2 μg/mouse approximates feasible human occupational exposures. Therefore, the results of this study indicate that caution should be used to limit human exposures to MWCNT.

## Background

The nanotechnology industry is a multibillion dollar industry and is expected to reach a trillion dollars by 2015 [1]. Carbon nanotubes are long thin nanoparticles that are composed of a single wall (SWCNT) or multiple walls (MWCNT) of graphene sheets rolled into tubes. MWCNT have potential applications in many consumer and industrial settings including medical devices, batteries, the automobile industry, electronic processes and the aerospace industry [2,3]. Carbon nanotubes are light and easily aerosolized making workplace exposure to nanoparticles a potentially significant source of human exposure. The material resists degradation and may persist in the body for extended periods of time [4,5]. The respiratory tract is a likely route of exposure due to the low density and small size of airborne nanoparticles. Similar to inhaled asbestos fibers, MWCNT deposited in the lungs of mice by pharyngeal aspiration or inhalation produced histologic changes including inflammation and fibrosis as well as hypertrophied and hyperplastic bronchiolar and alveolar epithelial cells [4,6-8]. Additional changes in some alveolar epithelial Type II cells of MWCNT-exposed mice include cellular atypia [8]. MWCNT can reach the alveolar region, the interstitium and the pleural space after both aspiration and inhalation [8-10]. Some macrophages that contain MWCNT particles have been observed without nuclei and with MWCNT connecting dividing chromosomes indicating that carbon nanotubes may be capable of inducing errors in cell division *in vivo* following either aspiration or inhalation exposure [8]. Type II cells from rodents exposed to MWCNT been shown to have micronuclei, indicating either a higher level of chromosome damage or mitotic spindle disruption [11]. *In vitro* investigations have demonstrated that carbon nanotubes disrupt the cell division apparatus and induce errors in chromosome number [11-14].

The multistage nature of cancer has been described in liver, skin and mammary as well as lung models for cancer [15-18]. Carcinogenic agents can act in one or all of the stages of the neoplastic process. Initiating agents typically cause a heritable change in DNA while tumor promoters induce proliferation of DNA damaged cells to form visible preneoplastic or benign clones [19]. During the last stage of neoplastic development (progression) malignant characteristics, karyotypic instability and frank neoplasms appear [19]. A complete carcinogen can act at all three stages. Cellular proliferation is a feature of the second phase of pulmonary carcinogenesis (promotion) [16,17]. Of interest, epithelial hyperplasia and cellular atypia were observed in mice exposed to MWCNT *in* vivo [8]. Therefore, the potential for carcinogenicity is of particular concern. In addition, previous studies have indicated the potential for carbon nanotubes to act during the progression of cells from preneoplastic and early benign lesions to carcinoma as shown by their ability to disrupt the mitotic spindle and induce chromosome alterations [17,19]. Investigations of MWCNT carcinogenicity have demonstrated that intraperitoneal or intrascrotal injection of MWCNT results in mesotheliomas in p53 +/- transgenic mice and Fischer rats, respectively [20,21]. The high-dose and agglomeration of the 3 mg MWCNT exposure used in the Takagi et al. study have been questioned, since it resulted in a high death rate due to gastrointestinal occlusion [22]. However, a more recent study demonstrated the induction of mesothelioma after intraperitoneal injection of as little as 3 μg of MWCNT in mice [23]. Because the physical properties of MWCNT make respiratory exposure likely during the production and processing of commercial products and pulmonary exposures in rodents have indicated a potential for genotoxicity, inflammation, cell proliferation, cellular atypia, and migration to the pleural space in a manner similar to other long thin fiber-like materials that are carcinogenic, there is an urgent need to examine the potential for cancer in an animal model following inhalation of carbon nanotubes.

The overall objective of this study was to determine whether inhalation of MWCNT produced lung tumors in adult, male B6C3F1 mice using a two-stage, initiation-promotion protocol. The B6C3F1 mouse is the strain used by the National Toxicology Program to evaluate chemicals for potential carcinogenicity [24]. The B6C3F1 hybrid is of intermediate susceptibility for spontaneous lung tumor formation; however, the strain is less sensitive than the sensitive/intermediate 020 and BALB/cByJ strains [25,26]. In addition, there is a wealth of information on the spontaneous tumor response and lifespan of the B6C3F1 mouse strain [25,27-29]. This is the first investigation to examine the potential carcinogenicity of carbon nanotubes using a multi-stage carcinogenesis model in the B6C3F1 mouse lung.

## Results

### Foreign material observed during histopathology assessment

**Figure 1:**
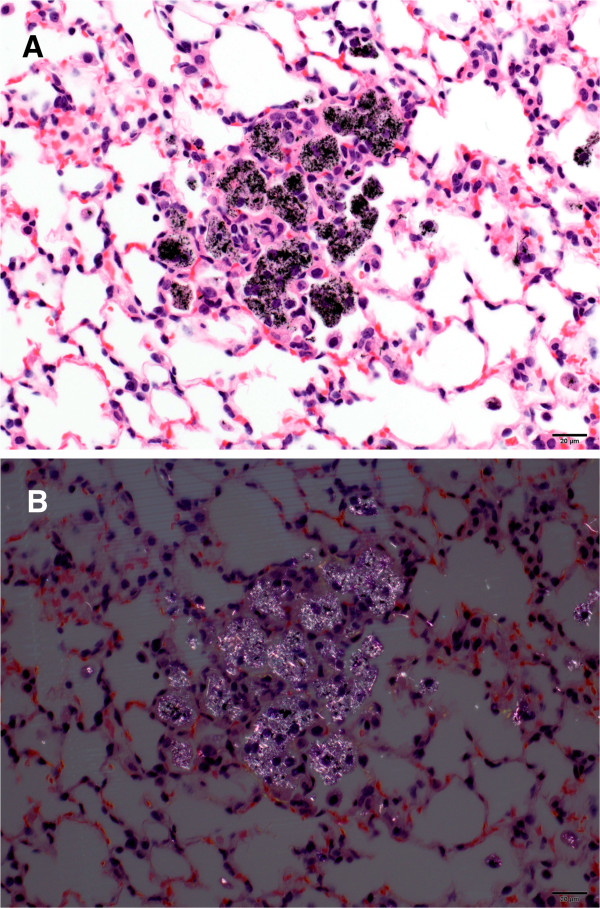
**Foreign material observed by light microscopy (A) and polarized light (B). A.** The figure is a low magnification image of MWCNT deposition in the lungs. Macrophages in alveolar spaces of the right apical lobe of the lung contain intracytoplasmic, black, elongate to finely granular particulate material (presumptive MWCNT). (40x). **B.** Using polarized light, the low magnification image demonstrates presumptive MWCNT are seen in macrophages in the right apical lung lobe of mouse treated with MWCNT. The material in the macrophages are birefringent. (40x).

The initial MWCNT lung burden of the mice exposed to MWCNT was determined to be 31.2 ± 0.9 μg MWCNT/lung. Light microscopic analysis demonstrated foreign material (presumptive MWCNT) in the lungs of all mice in the MWCNT and MCA + MWCNT groups but not in MCA or Air control animals 17 months following exposure (Table 1). By light microscopy, the foreign material was approximately 0.5 to 5 microns in length, finely granular to elongated, blocked light with transmitted light (appeared black) and had bright whitish birefringence under polarized light (Figure 1A,B). Commonly seen in the cytoplasm of cells at terminal bronchioles and alveolar ducts, foreign material was either in presumptive macrophages or epithelial cells lining the airways, or in macrophages within connective tissue adjacent to airway epithelium (Table 1). It was also present in macrophages that formed occasional random small clusters in airways or alveoli, and were seen extracellularly in connective tissue and between cells. The diagnostic term “foreign material” was exclusively used to indicate the presumptive test article (MWCNT).

**Table 1:** Incidence of focal adenomatous hyperplasia, macrophage infiltration and foreign material in the lung by light microscopy

**Focal alveolar adenomatous hyperplasia, macrophage infiltration and foreign material observed in MWCNT–treated mice 17 months following exposure**				
	**Air**	**MCA**	**MWCNT**	**MCA + MWCNT**
Number of animals/group	56	54	49	42
Number animals with positive foreign material	0	0	49*	42*
Macrophage infiltration	3	9	32*	39*
Number animals with positive for focal adenomatous alveolar hyperpasia	7	8	14*	26*

Statistical significance at *p < .0001.

### Enhanced darkfield imaging of MWCNT

**Figure 2:**
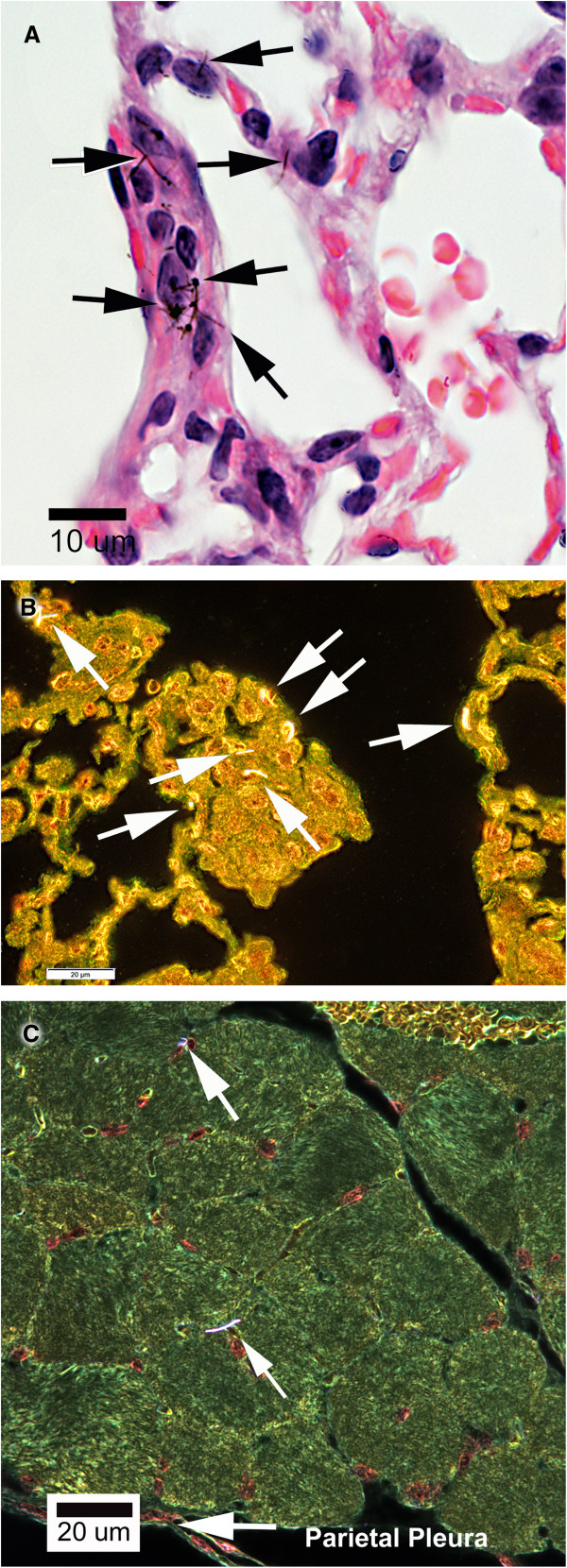
**Cytoviva imaging. A.** The figure is an example of typical images from light and enhanced darkfield imaging of MWCNT in lungs and diaphragm. The light micrograph of H&E stained section demonstrates MWCNT (black fibers) in the alveolar interstitium of a MWCNT Air exposed animal 17 months following inhalation exposure. This micrograph shows an example of MWCNT present within the alveolar interstium. Light microscope image using 100x oil immersion objective. Magnification bar is 10 microns. **B.** The micrograph is an enhanced darkfield image from the lung of an animal 17 months following exposure to MWCNT. The central area of the micrograph shows a region of alveolar wall with numerous MWCNT fibers present. The MWCNT are bright white due to imaging of scattered light over a broad range of wavelengths by this nanomaterial. Lung tissue, which does not significantly scatter light, is brown-to-orange and airspaces are black. The enhanced darkfield microscope image was photographed using a 100x oil immersion objective. The magnification bar is 20 microns. **C.** An enhanced darkfield imagine showing MWCNT in the diaphragm. MWCNT (indicated by the upper two arrows) are bright white. Nuclei are brown-to-orange, muscle cells are green and red blood cells are yellow. The parietal pleural border is indicated by the arrow in the lower part of the figure. Enhanced darkfield microscope image using 100x oil immersion objective. Magnification bar is 20 microns.

Imaging using CytoViva technology demonstrated MWCNT fibers in the lungs of MWCNT-exposed mice thus confirming the presence of MWCNT material observed by light microscopic analysis in the mouse tissues 17 months following exposure to the material. MWCNT material was observed by light microscopy in the interstitium of the lung (Figure 2A). With enhanced darkfield imaging MWCNT appear as bright fibrous structures. MWCNT were observed in alveolar tissue as shown in Figure 2B. MWCNT were also present within the alveolar macrophages (data not shown). In addition, enhanced darkfield analysis demonstrated MWCNT in the diaphragm (Figure 2C).

### Hyperplasia and macrophage infiltration in the lung

**Figure 3:**
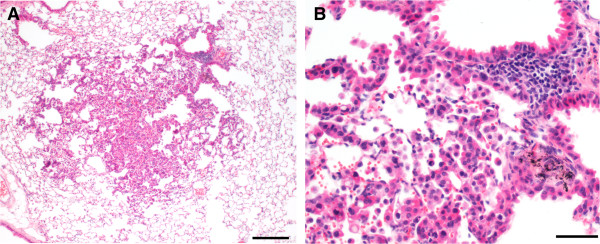
**Hyperplasia. A** Focal adenomatous hyperplasia in a mouse exposed to i.p corn oil and inhaled MWCNT. **A.** At low magnification, a focus of bronchoalveolar hyperplasia forms a discrete, hypercellular focus that retains normal alveolar architecture (bar = 200 microns). **B.** At higher magnification, the hypercellularity is attributable to a population of hypertrophied epithelial cells characterized by moderate anisokaryosis and mild karyomegaly. To distinguish these foci from foci of reactive hyperplasia and because of morphologic similarities to atypical adenomatous hyperplasia in the human lung, we have used the term focal adenomatous hyperplasia for these foci. MWCNT were sometimes seen within or near these foci (bar = 50 microns).

Regenerative alveolar epithelial hyperplasia is a common reaction of the murine lung to inhaled toxicants, including particles, while primary alveolar epithelial hyperplasia is believed to be a preneoplastic change . In humans, the form of primary bronchoalveolar hyperplasia considered preneoplastic is known as atypical adenomatous hyperplasia [30-33]. Therefore, in this paper, we have designated foci of marked, focal alveolar epithelial hyperplasia resembling human atypical adenomatous hyperplasia as focal adenomatous hyperplasia. Focal adenomatous alveolar hyperplasia was characterized by increased numbers of crowded alveolar epithelial cells that outlined contiguous alveolar septa in discrete, generally random locations (Figure 3). Animals with foci of focal adenomatous hyperplasia and macrophage infiltration were noted. The number of animals or incidence of focal adenomatous alveolar epithelial hyperplasia, macrophage infiltration, foreign material, and multifocal adenomatous bronchiolo-alveolar hyperplasia in the terminal bronchiole/alveolar duct regions were increased in both groups exposed to MWCNT (MCA+ and MCA-). The incidence of focal adenomatous hyperplasia was greatest in the MCA + MWCNT group relative to MWCNT, MCA and Air groups (Table 1). Focal alveolar epithelial hyperplasia was scored as marked (adenomatous) in 2%, 2%, 5%, or 27% of mice in the air, MCA, MWCNT, or MCA + MWCNT groups, respectively.

Incidences of macrophage infiltration were higher in the MWCNT or MCA + MWCNT groups relative to the air or MCA groups (Table 1). It was seen as occasional, random, small clusters of macrophages in airways or alveoli, or as slightly increased numbers in interstitial connective tissues often near terminal bronchioles or alveolar ducts.

### Lung adenoma and adenocarcinoma

The incidences, or number of mice with tumors, of bronchiolo-alveolar adenoma, bronchiolo-alveolar adenocarcinoma, and their combined incidence was greatest in the MCA + MWCNT group relative to the other groups for each individual lung lobe and for the entire lung considered as a single tissue (Table 2).

**Table 2:** Incidence and multiplicity of lung tumors in B6C3F1 mice 17 months following exposure to Mitsui-7 MWCNT

**Treatment**	**Total number of mice**	**Percent of animals with tumors**	**Total number of lung tumors**	**Lung tumors/total number of animals**	**Number of adenocarcinomas/group**
**AIR**	56	23.2	17	0.25 ±0.166	7
**MCA**	54	51.9*	41*	0.81 ±0.167	14*
**MWCNT**	49	26.5	20	0.38 ±0.185	7
**MCA + MWCNT**	42	90.5*	133*	2.9 ±0.386*	56*

*Indicates statistical significance at p < .0001 ± indicates standard deviation.

**Figure 4:**
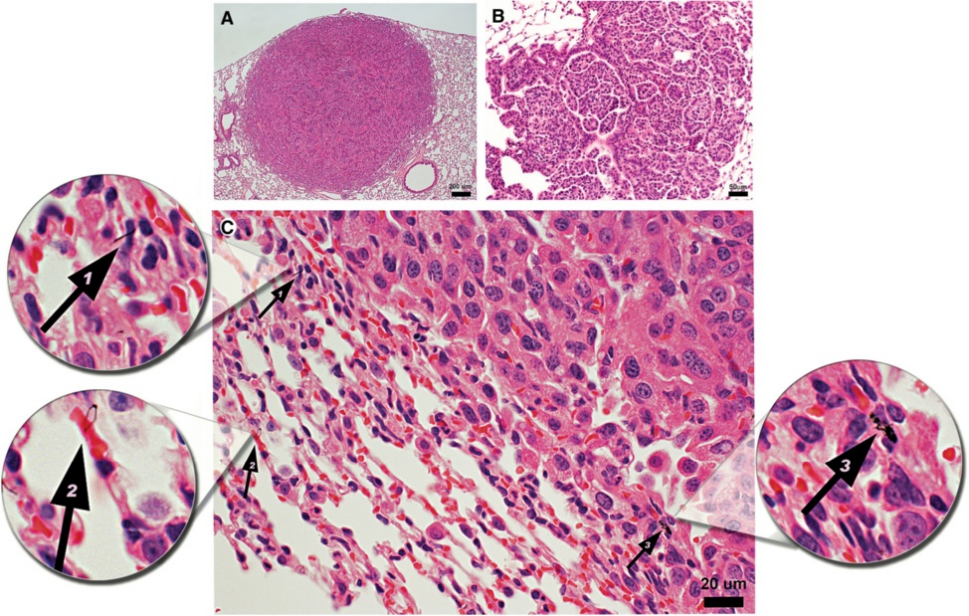
**A, B and C Lung adenoma, 20× and 40×. A.** The figure is a representative pulmonary bronchio-alveolar adenoma from a male B6C3F1 mouse treated with MCA + MWCNT. The mass is composed of relatively uniform cells that compressed the surrounding lung tissue. The photo with taken with a 4x objective. The magnification bar is 200 microns. **B.** Figure 4**B** is a photomicrograph of a pulmonary bronchiolo-alveolar adenoma from a MCA + MWCNT treated mouse. The focal, moderately cellular mass involved a bronchiole. The mass was composed of relatively uniform cells that distorted and replaced alveolar architecture. The image was photographed using a 20x objective. The magnification bar is 50 microns. **C.** The light micrograph is a higher magnification of the H&E stained section demonstrating MWCNT (black fibers) in the tissue surrounding the bronchiolo-alveolar adenoma (arrows 1, 2 and 3). The light microscope image was taken using 100x oil immersion objective. The magnification bar is 20 microns.

Bronchiolo-alveolar adenomas were focal, densely cellular, slightly compressive masses that distorted alveolar architecture and replaced alveolar spaces (Figure 4). The masses were composed of proliferative epithelial cells that formed irregular papillary structures, ribbons, or solid clusters separated by delicate, fibrovascular stroma. The cells were polygonal, moderately uniform in size, and had small to moderate amounts of eosinophilic, occasionally vacuolated cytoplasm. Nuclei were small, round to oval, moderately uniform with inconspicuous nucleoli, and mitoses were few to absent. At terminal sacrifice, the percent of mice (incidence) with bronchiolo-alveolar adenomas in the MCA followed by air and MCA + MWCNT groups were 33% and 76%, respectively, exceeding the air (11%) and MWCNT (18%) groups. The NTP has reported a range of 2-30% lung adenomas in vehicle control B6C3F1 male mice thus indicating that the mice in the current study have a spontaneously-occurring lung adenoma incidence within the range expected in this mouse strain (Table 3) [24]. Furthermore, the morphology of bronchiolo-alveolar adenomas in the groups that received MCA and/or MWCNT did not differ appreciably from the spontaneously occurring neoplasms in the air group.

**Table 3:** Incidence of lung tumors in early euthanized mice and mice 17 months after exposure

**Euthanasia**	**Early sacrifice**				**Terminal sacrifice**			
	**Air**	**MCA**	**MWCNT**	**MCA + MWCNT**	**Air**	**MCA**	**MWCNT**	**MCA + MWCNT**
**Number of animals**	**4**	**6**	**6**	**13**	**56**	**54**	**49**	**42**
**Number of Bronchiolo-alveolar adenomas**	**0**	**1**	**0**	**4**	**6**	18*	**9**	32*
**% of mice with one or more Bronchiolo-alveolar adenoma**	**0%**	17%*	**0%**	31%*	**11%**	33%*	**18%**	76%*
**Number of Bronchiolo-alveolar adenocarcinomas**	**0**	**1**	**0**	**7**	**7**	**12**	**7**	**26**
**% of mice with Bronchiolo-alveolar Adenocarcinomas**	**0%**	17%*	**0%**	54%*	**13%**	22%*	**14%**	62%*
**Number of mice with one or more– Bronchiolo-alveolar adenoma and/or Bronchio-alveolar adenocarcinomas**	**0**	**2**	**0**	**8**	**13**	28*	**13**	38*
**Percent of mice with lung tumors**	**0%**	**33%**	**0%**	62%*	**23.2%**	51.9%*	**26.5%**	90.5%*

*Indicates statistical significance at p < .0001.

**Figure 5:**
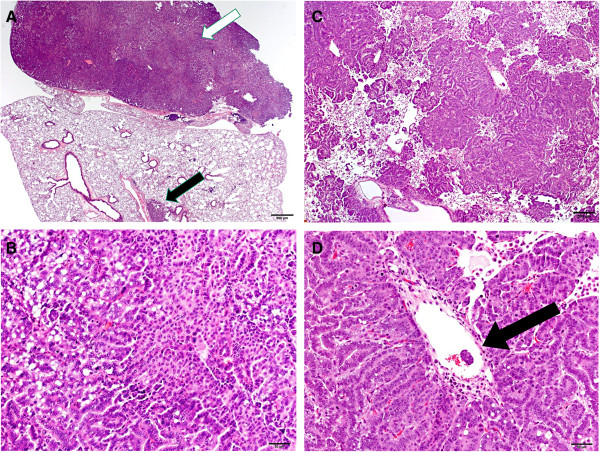
**Pulmonary bronchiolo-alveolar adenocarcinomas in MCA + MWCNT treated mice. A.** The photomicrograph shows a right cardiac lung lobe of a MCA + MWCNT-treated mouse that contains a bronchiolo-alveolar adenocarcinoma (white arrow). A bronchiolo-alveolar adenoma (black arrow) is in the adjacent lung lobe. (2x). The magnification bar is 500 microns. **B.** The figure is a photomicrograph of a bronchiolo-alveolar adenocarcinoma in the right cardiac lobe of a mouse lung treated with MCA + MWCNT (20x). This infiltrative adenocarcinoma filled ~85% of the lobe on histologic cross section. The scale bar is 200 microns. **C.** The photomicrograph is a higher magnification of the bronchiolo-alveolar adenocarcinoma of the right cardiac lobe in figure **B** showing heterogeneous growth and pleomorphic cytologic features (40x). The scale bar is 50 microns. **D.** The photomicrograph shows a metastasis of the bronchiolo-alveolar adenocarcinoma in the right cardiac lobe of a mouse lung 17 months following treatment with MCA + MWCNT (40×). The arrow demonstrates a metastasis in a pulmonary vein. The scale bar is 50 microns.

In contrast to bronchiolo-alveolar adenomas, bronchiolo-alveolar adenocarcinomas had increased cellular atypia, higher nuclear to cytoplasmic ratios, and larger nucleoli (Figure 5A). Several cytologic patterns were often present within the same mass, and included ribbons, papillary structures, or solid clusters (Figure 5B and 5C). The incidences of bronchiolo-alveolar adenocarcinomas in the MCA and MCA + MWCNT groups were 22% and 62%, respectively, exceeding the air (13%) and MWCNT (14%) groups (Table 3). The incidence of bronchiolo-alveolar adenocarcinoma in the MCA + MWCNT group (62%) greatly exceeded the NTP historical vehicle control range of 4-24% for male B6C3F1 mice [24]. The combined incidences of bronchiolo-alveolar adenoma and bronchiolo-alveolar adenocarcinoma in the MCA or MCA + MWCNT groups were 51.9% and 90.5%, exceeding the air (23.2%) and MWCNT only (26.5%) groups, and exceeding or greatly exceeding the NTP historical vehicle control range for male B6C3F1 mice (14-40%), respectively [24].

**Figure 6:**
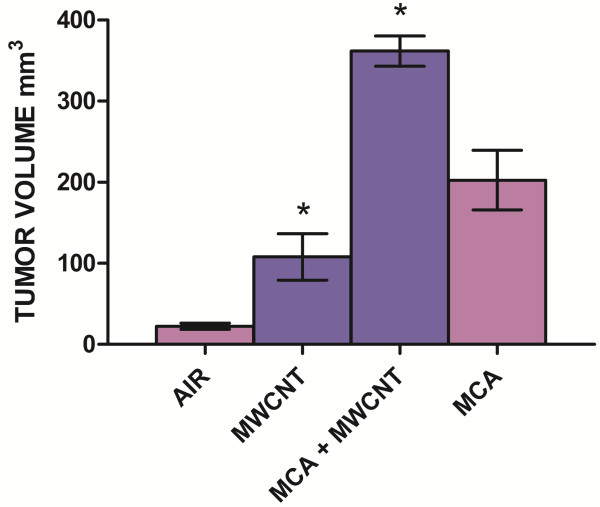
**Volume of lung tumors 17 months after exposure to MWCNT.** The figure demonstrates the volume of the lung occupied by tumor per individual mouse. The volume of the lung occupied by tumor was greater in the MCA + MWCNT (361 mm^3^+/-18.72) compared to the mice treated with MCA (202.61 mm^3^+/-36.75). The MWCNT group had an average tumor volume of 107.88 mm^3^+/- 28.70 compared to the negative control, Air group, with a volume of 22.29 mm^3^ +/- 3.93. *Indicates a significant difference compared to the respective air control group at p < .0001.

The number of bronchiolo-alveolar adenomas and bronchiolo-alveolar adenocarcinomas was increased in the MCA and MCA + MWCNT groups relative to the Air or MWCNT groups (Table 3). The numbers of these neoplasms were greatest in the MCA + MWCNT group relative to the other groups. The MCA + MWCNT treated mice had a mean of 2.9 tumors/mouse compared to 0.81 in MCA, 0.25 air alone and 0.38 MWCNT-treated mice (Table 2). The volume of the lung occupied by tumor was greater in the MCA + MWCNT compared to the other groups (Figure 6). Three mice exposed to both MCA and MWCNT that were euthanized early had tumors with evidence of local invasion of the lung tissue (5.5%). Figure 5D demonstrates lung adenocarcinoma tissue invading a vein.

**Figure 7:**
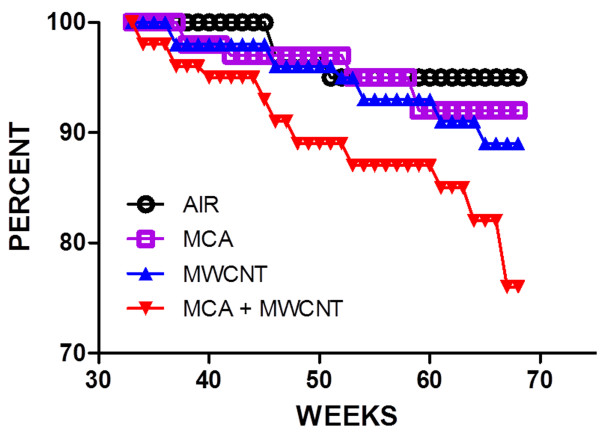
**Life table of mice treated with one dose of corn oil (Air), MC (MCA), MWCNT only MWCNT or MCA followed by MWCNT (MC + MWCNT).** The life table in Figure 7 shows the percent of mice alive in each treatment group at 30 weeks after exposure to the time of sacrifice 70 weeks after exposure. The MCA + MWCNT treated animals had a greater number of early deaths than the MWCNT, the MCA or the Air treated mice.

Animals that were euthanized early were analyzed separately from the mice that were euthanized 17 months after exposure (Table 3). Four air control mice were euthanized early due to morbidity. Specifically, these air-exposed control mice were terminated due to a 20% loss of their body weight. One of the four air controls had a dental tumor at euthanasia. No other lesions or lung tumors were observed in the air mice either grossly or by pathological analysis. Six of the animals exposed only to MWCNT were euthanized early due to morbidity. These mice were euthanized due to significant weight loss and one animal was found to have an enlarged heart. Two of these mice had liver tumors. No lung tumors were observed in the early euthanized mice that were only exposed to MWCNT. Six animals treated with MCA followed by air were euthanized early due to a 20% or greater weight loss. One MCA mouse had a lung adenoma and one mouse had a lung adenocarcinoma. Thirteen of the animals treated with MCA followed by MWCNT were euthanized due to significant weight loss. Seven animals treated with MCA followed by MWCNT had lung tumors (Table 3). The life table demonstrates the time period of early deaths (Figure 7). The mean age of death of the animals that were euthanized early was as follows: 11.18 ± 2.08 months in the air group, 12.28 ± 0.72 months in the MCA group, 10.63 ± 1.27 months in the MWCNT treatment group and 11.22 ± 1.58 months in the MCA + MWCNT-treated group (Figure 7). There was not a significant difference in the age of death between groups.

### Serosal tumors

**Figure 8:**
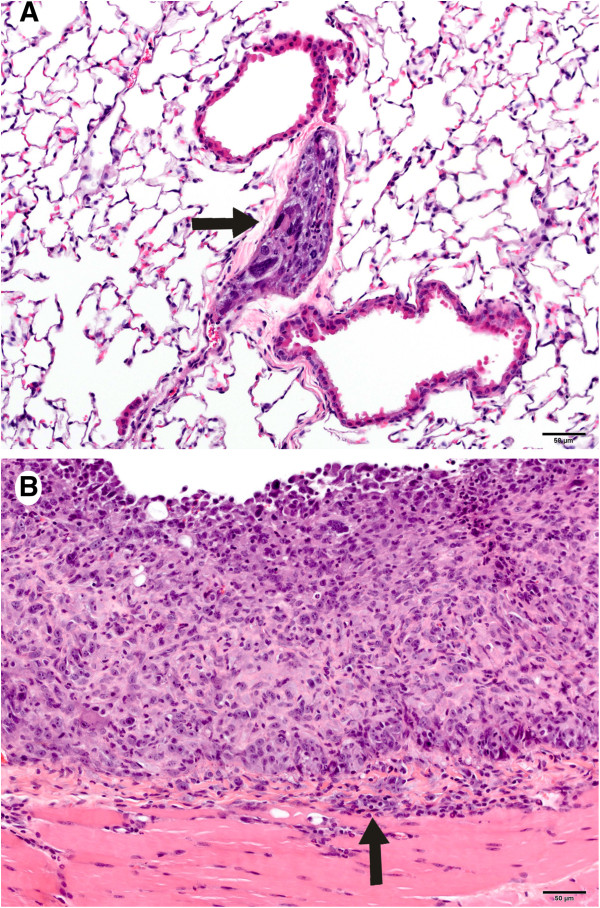
**Malignant serosal tumors in the lung (A) and the diaphragm (B) in mice exposed to MC followed by MWCNT. A.** The photomicrograph shows a pulmonary vein in the right cardiac lung lobe that contained variably-sized polygonal to spindloid cells similar to those on the diaphragm (Figure 8**B**). The arrow indicates a metastasis of the malignant serosal tumor (20×). The magnification bar is 50 microns. **B.** The skeletal muscle of the diaphragm in the photomicrograph is infiltrated by a nodular mass composed of variably-sized polygonal to spindloid cells (malignant serosal tumors). (20×). The magnification bar is 50 microns.

**Figure 9:**
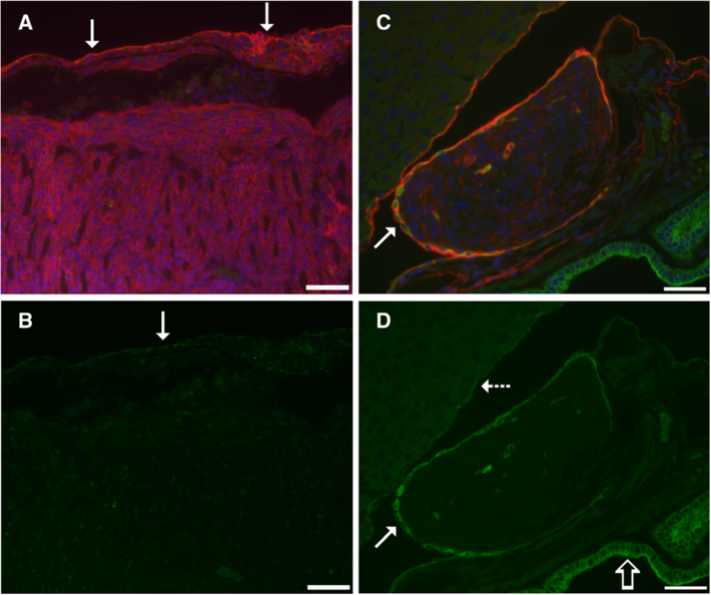
**Immunofluorescence staining for podoplanin (red) and cytokeratins (green) in malignant serosal tumors morphologically consistent with sarcomatous mesothelioma.** In **A**, the lining mesothelium (solid arrows) stained positively for podoplanin in this double label immunofluorescent image of diaphragm. The cells beneath the mesothelial lining are also red due to expression of podoplanin and these are cells of a malignant serosal tumor. In **B**, only the single label green fluorescence is shown to demonstrate weak expression of cytokeratins in the lining mesothelium (solid arrow), while staining of the subjacent tumor for cytokeratins is equivocal. In **C**, a double label immunofluorescent image demonstrates a malignant serosal tumor between the liver and gall bladder that is lined by reactive mesothelium (solid arrow) which stains red for podoplanin as well as green for cytokeratins. The cells of the subjacent malignant serosal tumor stain weakly red for podoplanin. In **D**, the photomicrograph shows this same tumor but only the green fluorescence for cytokeratins. The reactive mesothelium lining the malignant serosal tumor (solid arrow) stains green for cytokeratins while the serosal tumor has no evidence of cytokeratin expression. The normal mesothelium lining the liver is weakly positive for cytokeratins (dashed arrow). The epithelium lining the gall bladder (open arrow) strongly expresses cytokeratins. Magnification bar is 50 μm.

Malignant serosal tumors morphologically consistent with malignant sarcomatous mesotheliomas were seen in five mice (9%) in the MCA + MWCNT group and one mouse (2%) in the MCA group (Table 4, Figure 8). Consistent with the serosal dissemination of malignant mesotheliomas, multiple tissues were affected in four of the six affected mice. Spontaneous mesotheliomas in B6C3F1 mice are rare [24]. In recent years, a series of immunologic markers have been developed to augment the morphologic diagnosis of mesothelioma in man [34,35]. For that reason, we further characterized the serosal tumors by immunofluorescence (IF) for vimentin, cytokeratin and podoplanin in at least one tumor from each affected mouse. All presumptive sarcomatous mesotheliomas stained positively for podoplanin and vimentin. Podoplanin staining was strongly positive in all tumors evaluated from 5 mice and in one mouse varied from weakly to strongly positive in three different serosal tumors. Two wide-specrum cytokeratin antibodies were used because of the variable cytokeratin staining of sarcomatous mesotheliomas in man (Figure 9) [36,37]. Cytokeratin staining with a mouse anti-wide spectrum cytokeratin antibody was negative in four mice and equivocal in two. Cytokeratin staining with a rabbit anti-pancytokeratin antibody was negative in tumors from four mice and equivocal to faintly positive in tumors from two mice.

**Table 4:** Malignant serosal tumors in MCA + MWCNT Exposed Mice

	**MCA + MWCNT**					**MCA**
**Mesothelioma by animal #**	**76**	**245**	**261**	**301**	**309**	**192**
Epididymis		X		X	X	
Fat					X	
Liver			X			
Lymph node					X	
Bronchial lymph node				X		
Skeletal muscle diaphragm	X			X	X	X
Pancreas			X	X		
Mesentery	X		X	X	X	X
Lungs				X		

## Discussion

The primary objective of this investigation was to evaluate if exposure to aerosolized MWCNT promotes the growth of DNA damaged cells and/or is a complete carcinogen. To accomplish this, mice were exposed to aerosolized MWCNT (5 mg/m^3^, 5 hours/day) for 15 days. Initial MWCNT lung burden in these mice was 31.2 ± 0.9 μg MWCNT/mouse [38]. In order to evaluate the relationship of these MWCNT lung burdens to human MWCNT exposures, we compared the MWCNT lung burdens in these mice with potential human occupational exposures. OSHA has not yet established exposure limits for carbon nanotubes; however, MWCNT are regulated as respirable particulates not otherwise regulated (PNOR). The PNOR have an OSHA Permissible Exposure Limit (PEL) of 5 mg/m^3^[39]. NIOSH recently published a Current Intelligence Bulletin with a Recommended Exposure Limit (REL) of 1 μg/m^3^ for carbon nanotubes which is 5000-fold lower than the OSHA PNOR PEL [40].

Assuming a mouse alveolar epithelium surface area of 0.05 m^2^[41], the 31.2 μg MWCNT lung burden would result in 624 μg MWCNT/m^2^ alveolar epithelium. Using the alveolar epithelial surface area of 102 m^2^ for human, the equivalent human lung burden would be 63.6 mg [41].

If the MWCNT mass median aerodynamic diameter (MMAD) =1.5 μm were used, minute ventilation of 20 L/minute for a person performing light work [42] and an alveolar deposition fraction of 30% [43] (for 240 work days per year), the equivalent lung burden in workers exposed at the previous draft REL for CNT of 7 μg/m^3^ would be achieved in approximately 13 years [44]. This indicates that the mouse MWCNT lung burdens in this study approximate feasible human occupational exposures.

Inhalation of multi-walled carbon nanotubes (MWCNT) for 15 days following a single intraperitoneal injection of the known initiator MCA led to increased incidence and numbers of bronchiolo-alveolar adenomas and bronchiolo-alveolar adenocarcinomas in B6C3F1 male mice. The combined incidence of bronchiolo-alveolar adenomas and bronchiolo-alveolar carcinomas of 90.5% for the MCA + MWCNT group greatly exceeded that in groups of mice exposed to air or MCA and the NTP historical vehicle control range for B6C3F1 male mice [24]. Additionally, the numbers of bronchiolo-alveolar adenomas or bronchiolo-alveolar adenocarcinomas were greatest in the MCA + MWCNT group compared to other groups. The data demonstrate that MWCNT may act as a carcinogen that promotes the growth of initiated lung cells, resulting in the development of lung adenocarcinoma.

The strong tumor promotion that was observed in the current study may have resulted from a combination of effects that have been observed following exposure to carbon nanotubes. MWCNT material was observed in the diaphragm and in the lungs, both within macrophages and the interstitium. MWCNT are internalized by macrophages following pulmonary exposure. In addition, MWCNT material has been observed in the interstitium. MWCNT exposure has been shown to induce fibrosis as early as seven days post-exposure [8,10,45]. In previous experiments, the post-exposure pulmonary distribution, pulmonary fibrotic response and transport of MWCNT to systemic organs were examined at various times post-exposure, from 1 to 336 days [46,47]. Fibrillar collagen in the lungs was specifically stained and the quantity of fibrillar collagen in the alveolar region was measured by morphometry. These measurements of fibrillar collagen in the alveolar region of the lungs demonstrated a fibrotic response to inhaled MWCNT which was significantly above vehicle controls and progressively increased throughout the 336 days post-exposure study period [46]. These studies have demonstrated that inhaled MWCNTs are deposited throughout the alveolar region of the lungs and are retained in the alveolar tissue. Additionally MWCNT were demonstrated in the visceral pleura, pleural space and parietal space [9]. MWCNT that penetrate the visceral pleural induce pleural inflammation and cell proliferation in a manner similar to asbestos [8,48,49]. MWCNT have further been shown to penetrate the cytoplasmic membrane and nuclear envelope [50,51].

Asbestos and MWCNT also induce inflammation, fibrosis, cell proliferation and cellular atypia in the lung [8,48,49]. Cell proliferation and inflammation are important events in the promotion of cancer [16,17,52-55]. Indeed in the current study, inhaled MWCNT induced dramatic hyperplasia and a moderate increase in adenomas however, the increase in adenomas was not statistically significant. In addition, MWCNT exposure did not result in an increased number of adenocarcinomas. The significant hyperplastic response that was observed after exposure to MWCNT material without prior initiation indicates that the material was a tumor promoter. The dramatic increase in adenomas and adenocarcinomas after MCA initiation followed by MWCNT-exposure demonstrate that inhaled MWCNT material is a strong tumor promoter. Strong tumor promoters increase the growth of chemically initiated as well as spontaneously initiated cells [56,57]. Although the data of the current investigation do not indicate that inhaled MWCNT material act as tumor initiators, the data demonstrate the strongest promotion response observed in the lung using occupationally relevant material [15,52,58-60]. The data further indicate that MWCNT may initiate lung responses similar to the carcinogenic fiber asbestos [4,61-63].

The dimensions of the nanotubes as well as their surface properties are important in the inflammatory response. Pulmonary exposure to SWCNT and MWCNT causes inflammation and fibrosis; however, the inflammatory response following MWCNT exposure is more pronounced than the response observed following SWCNT exposure [8,64,65]. The degree of inflammation resulting from asbestos and MWCNT is determined by the diameter and the length [4,66,67]. Carbon nanotubes of approximately 50 nm in diameter cause more inflammation than nanotubes of less than 20 nm or greater than 150 nm [67]. In addition, the rigid MWCNT of 40–50 nm diameter and at least 4 microns long were the most inflammatory [67,68]. Although these studies indicate that the diameter and length of carbon nanotubes may alter MWCNT-induced carcinogenicity further investigations are required to fully characterize the role of the dimensions as well as the physical properties in the carcinogenic response.

Classical multistep carcinogenesis models involve initiation, promotion and progression. Exposure to a genotoxic agent initiates a population of genetically altered cells which expand in number through the action of promoters and undergo additional genetic changes during the progression process [16,17,69]. Several studies suggest both genotoxicity and promotion from the classical carcinogenic high aspect ratio particle, asbestos. Oxidant generation from inflammation has been shown to damage the DNA and can initiate cancer [54]. Asbestos and carbon nanotubes have been shown to induce disruption of the cell division apparatus and errors in chromosome number (aneuploidy) *in vitro*[12,14,70]. The long, thin asbestos fibers of less than 0.25 μm diameter and at least 5 microns in length are the most genotoxic [71,72]. The mutagenicity of asbestos fibers is correlated with the potency as a carcinogen [73]. Evidence from epidemiological studies has demonstrated that asbestos can act as a tumor promoter at low doses as well as a tumor initiator at longer and/or higher exposure levels [74]. In several human epidemiology studies, smoking exposure and asbestos interact in a more than additive fashion in causing lung cancer [75-79]. There are multiple mutagens in cigarette smoke that have the potential to initiate cancer [80]. Humans are also potentially exposed to many other mutagens that could initiate cancer such as radon, polychlorinated biphenyls, hexavalent chromium, naphthalene and benzo-a-pyrene in diesel exhaust [81-85]. Thus, it is plausible to suggest MWCNT could potentially act as promoters in individuals who smoke or are exposed to other initiators.

Previous studies to examine rodent exposure to asbestos by inhalation or pharyngeal aspiration have shown that asbestos is carcinogenic in the rat lung by this route but only weakly positive in the mouse [86-88]. The data demonstrating that asbestos induces mitotic spindle disruption and aneuploidy would suggest that asbestos would be a strong tumor promoter; however, asbestos has not been administered in an initiation/promotion protocol in a mouse model. Although lung cancer has not been observed in either rats or mice following the intraperitoneal injection of asbestos or carbon nanotubes, mesothelioma has been reported. Abdominal or scrotal injection of mice with asbestos or long thin MWCNT of at least 3.9 micron in length and 50 nm in diameter caused mesotheliomas in p53 +/- transgenic mice and Fischer rats [20,21]. Recent investigations demonstrated that intraperitoneal injection of as little as 3 μg of MWCNT in genetically modified mice (p53+/-) induced mesothelioma [23]. By contrast, an IP exposure of Wistar rats to short MWCNT of < 1 micron in length resulted in mesothelioma in 5/150 MWCNT-exposed animals but those findings were not statistically significant due to a high peritoneal mesothelioma rate in the control group [89]. The high background rate of peritoneal mesotheliomas (1/26) is unusual for the Wistar rat [89-91]. A subsequent study demonstrated that high exposures (1 and 10 mg/rat) of thin, rigid MWCNT by intraperitoneal injection caused mesotheliomas (54). When the diameter of the nanotubes was considered, MWCNT of 50 nm in diameter were more carcinogenic than nanotubes of less than 20 nm or greater than 150 nm [67]. These findings suggest that the diameter and length are critical in the carcinogenic response to MWCNT, a finding that is similar to classical studies of asbestos fiber carcinogenicity [92].

A limitation of the current study is that suitable non-carcinogenic particle controls do not exist in this model. We considered using the short multi-walled carbon nanotubes investigated by Muller et al., as a potential negative control [89]. However, since the interpretation of the Muller et al. study is affected by the unusual high background rate of peritoneal mesothelioma in the control group, this particle cannot be considered a confirmed negative control nor could we identify any carbon nanotube as a confirmed negative particle control for a carcinogenicity study. The identification of a suitable negative control nanotube will require further carcinogenicity studies that have yet to be published.

Malignant mesothelioma in humans has three major histologic patterns: epithelial, sarcomatous (sarcomatoid), and biphasic [93,94]. Using standard histopathology, the major differential diagnoses for malignant mesothelioma include broncho-alveolar adenocarcinoma of the lung, metastatic carcinoma and metastatic sarcoma [94]. The diagnosis of malignant mesothelioma in humans can be supported by staining for proteins commonly expressed in mesotheliomas, including calretinin, cytokeratins, mesothelin, WT-1 and podoplanin (D2-40) [34]. Podoplanin is among the markers most consistently expressed in human malignant sarcomatous mesotheliomas [34,95]. The malignant serosal tumors seen in the mice in our study consistently expressed podoplanin. However, the staining for cytokeratins was negative to equivocal, a finding that is also sometimes seen in sarcomatous mesothelioma in humans [35,36]. One review noted that only 13% of human sarcomatous mesotheliomas were positive for cytokeratin 5/6 and none were positive for seven other epithelial markers [37]. However, in one study using a cocktail of mouse anti-human monoclonal antibodies, 93% of the cases of human sarcomatous mesotheliomas demonstrated cytokeratin expression [96]. However, there are protein sequence differences between human and mouse cytokeratins. In addition, even with blocking steps, indirect immunohistochemistry using mouse antibodies on mouse tissues results in some degree of binding of the secondary anti-mouse IgG antibody with endogenous IgG located in the mouse tissue. It is for this reason that we used both a mouse monoclonal antibody and a rabbit anti-pancytokeratin antibody to stain for cytokeratins in this study. However, the negative to equivocal staining of the serosal tumors for cytokeratins in this study should be interpreted with an understanding that mesotheliomas are very rare in the mouse and that techniques for identifying mesothelioma markers in mice are not as advanced as they are for identifying those markers in human tissue. The negative staining does not mean that there are no cytokeratins in the serosal tumors of our study, only that no cytokeratins could be identified with the antibodies used in this study.

In humans, podoplanin staining in the absence of cytokeratin staining can be seen in several different sarcomas as well as in malignant mesothelioma [34,97]. The malignant serosal tumors seen in this study were morphologically consistent with malignant sarcomatous mesotheliomas with five of the six tumors involving multiple peritoneal serosal surfaces. The remaining malignant serosal tumor was limited to the male urogenital tract, a common site for mesothelioma in rats but not in control mice [98]. In humans, a diagnosis of cytokeratin negative sarcomatous mesothelioma is usually made by excluding other potential diagnoses [36]. Given the rarity of mesotheliomas in the mouse, we could not exclude other diagnoses with absolute certainty [24]. Thus, the characteristics of these tumors are consistent with, but not diagnostic of, mesothelioma. The principal differential diagnosis is pleural sarcoma.

These tumors are considered similar to the serosal tumors diagnosed as pleural sarcomas or malignant mesenchymal neoplasms in the classical asbestos studies in rats conducted by Stanton and co-workers who noted their comparability to human mesotheliomas [92].

## Conclusions

This study is the first to demonstrate that inhalation exposure to some MWCNTs promotes the growth of initiated lung cells in a wildtype mouse. Ninety percent of the mice exposed to MCA followed by MWCNT had lung adenocarcinoma and adenomas (mean of 2.9/mouse) compared to 23% of the filtered air controls (mean of 0.25/mouse), 26.5% of the MWCNT-exposed (mean of 0.38/mouse), and 51.9% of the MCA followed by air-exposure (mean of 0.81/mouse). The data therefore demonstrate that inhaled MWCNT are strong promoters of pulmonary adenomas and adenocarcinomas in B6C3F1 mice. Furthermore, the strong tumor promotion response observed following exposure to MWCNT was observed in a hybrid mouse that is intermediate in sensitivity to lung cancer [25,26]. Because this B6C3F1 hybrid is used by the NTP to determine potential carcinogenesis, the study can be compared to a wealth of historical data generated by the NTP [24].

Furthermore, the current investigation suggests that inhaled MWCNT can promote the growth of malignant serosal tumors consistent with sarcomatous mesothelioma. MWCNT inhalation increased the incidence from 2% in the MCA exposed mice to 9% in the MCA + MWCNT, a 4.5 fold increase. However, malignant serosal tumors are uncommon tumors in mice and their potential promotion by MWCNT is of concern. NIOSH is undertaking an inhalation exposure designed to further evaluate the potential for MWCNT to cause mesothelioma. The mouse MWCNT lung burdens in the investigation are relevant to feasible human occupational exposures. While extrapolation to human health is premature, humans working with MWCNT may be exposed to numerous tumor initiators in the course of their daily lives. Results from this study suggest that caution should be taken during production and processing to limit human inhalation exposures to MWCNT.

## Methods

### MWCNT

MWCNT used in this study were obtained from Hodogaya Chemical Company (Mitsui-7 MWNT-7, lot #061220-31) and were manufactured using a floating reactant catalytic chemical vapor deposition method followed by high temperature thermal treatment in argon at 2500°C using a continuous furnace [99]. The bulk material was characterized by high-resolution transmission electron microscopsy under a Philips CM 20 transmission electron microscope (TEM) with an EDS (EDAX/4p1) as described previously [100]. MWCNT trace metal contamination of 1.32% with iron being the major metal contaminant was 1.06% [101].

### MWCNT inhalation exposure and aerosol characterization

The MWCNT aerosol was generated using an acoustical-based computer controlled whole body inhalation system designed and constructed by McKinney et al, [100]. In brief, the inhalation exposure system combines air flow controllers, aerosol particle monitors, data acquisition devices, and custom software with automated feedback control to achieve constant and repeatable exposure chamber temperature, relative humidity, pressure, aerosol concentration, and particle size distributions. The generator produces airborne particles continuously for long periods of time with minimal fluctuations during an exposure period. The uniformity of test atmosphere in the chamber was evaluated to have a total variation of <5%. In this study, the MWCNT aerosol mass concentration was continuously monitored with a Data RAM (DR-40000 Thermo Electron Co, Franklin, MA), and gravimetric determinations (37 mm cassettes with 0.45 μm pore-size Teflon filters) were used to calibrate and verify the Data RAM readings. Chen et al. have provided a detailed characterization of MWCNT samples taken from the animal exposure chamber [102]. In addition, cascade impactors (MOUDI, Models 110 and 115, MSP Co., Shoreview, MN) were used to determine the mass-based particle size distributions by fractionating the particles into 15 size fractions ranging from 10 nm to 18 μm. The mass median aerodynamic diameter was determined to be 1.59 μm and geometric standard deviation of 1.69. The count mode aerodynamic diameter was 0.42 μm [101,102]. The target concentration of the mouse exposure was 5 mg/m^3^ for a duration of 5 hours/day for 15 days, with an accumulative exposure dose of 375 mg/m^3^ × hr. The values of detailed exposure parameters were presented in Additional file 1: Table S1. Based on data from Data RAM and filter samples, the mean concentrations among the 5 exposures were consistent at 4.6-4.7 mg/m^3^ with a daily variation between 4–8%. Depending on the concentration measured daily, the exposure time was adjusted accordingly to result in the target dose of 375 mg/m^3^ × hr. The mean exposure time per day was 320–330 minutes with a daily variation between 1–3%. The accumulated dose was measured between 372 and 379 mg/m^3^ × hours and therefore was very close to the target dose of 375 mg/m^3^ × hr. In brief, the study was well conducted to fulfill the exposure design.

### Initiation promotion protocol

Six week old male B6C3F1 mice (Jackson Laboratories, Bar Harbor, ME) were housed singly in a polycarbonate ventilated cage with HEPA-filtered air. Male mice were selected for the investigation because the preliminary data showing dose response, proliferation of type II cells, cellular atypia and migration of particles following carbon nanotube exposure were gathered using male mice [7-9,103]. The mice were fed ad libitum with Harlan 7913 irradiated NIH-31 modified 6% rodent chow. The initiation, promotion protocol developed previously by Alvin Malkinson was followed [15]. After a one week acclimation period, mice (60/group) were randomly assigned to a treatment group. The mice were treated following a two stage (initiation-promotion) protocol. An initiation-promotion protocol involved the administration of a low dose of a DNA damaging agent (methylcholanthrene, MCA) followed by administration of a suspected carcinogen that would promote the growth of DNA damaged cells (MWCNT). All mice received a single dose of either MCA (10 μg/g BW, i.p.) or vehicle (corn oil). One week after receiving MCA, mice were exposed to MWCNT by whole body inhalation (5 mg/m^3^, 5 hours/day) or filtered air (controls) for 15 days. Mice were euthanized 17 months after exposure to allow time for tumor development. Mice were divided into five blocks with staggered test substance administration start and end dates.

Because animals developing lung tumors have non-specific symptoms but may develop general signs of pain and distress, animals were monitored weekly for overt signs of morbidity and changes in body weight. Animals with skin lesions, ruffled fur, lethargy, shaking, penis or anal prolapse, erratic movements or paralysis were closely monitored for further signs of distress. Animals that had a loss of 20% or greater of body weight, were hunched or developed hind leg paralysis were euthanized for morbidity prior to the terminal sacrifice.

### Foreign material in lung tissues

MWCNT burden determinations were made using a procedure previously developed with minor modification [38]. After euthanasia, lungs were removed and frozen at -80°C and preserved for further processing. The lung tissue was digested in 25% KOH/methanol (w/v) at 60°C overnight, followed by centrifugation at 16,000 × g for 10 minutes. The supernatant was removed; the remaining pellet was mixed with 50% HNO_3_/methanol (v/v), and incubated at 60°C overnight, followed by centrifugation (16,000 × g, 10 minutes). After centrifugation, the supernatant was removed, and the pellet was resuspended in 10% NP-40 (v/v) in dH_2_O, followed by 30 second sonication using cup horn sonicator. MWCNT standards were processed in parallel with the lung samples. The optical densities of the solutions were measured at 700 nm using a UV/visible spectrophotometer. Lung MWCNT content was determined from a standard curve.

### Necropsy, histopathology and tumor counts

Groups of mice were divided into five blocks with staggered test substance administration start and end dates. The lungs and any masses from mice euthanized early were noted and the tissues collected for pathological analysis. The mice euthanized early, due to signs of morbidity [8], were analyzed separately from animals that were sacrificed 17 months after exposure. Mice were euthanized with an overdose of ≥100 mg/kg bw pentobarbital and exsanguinated. The lungs were fixed by intratracheal perfusion with 1 ml of 10% neutral buffered formalin. The mice were then necropsied following standard techniques [45]. Masses and lesions seen grossly were recorded on individual animal necropsy records (IANRs). The length, width and height of masses were measured in the MCA, air, MWCNT and MCA + MWCNT-treated mice, using a digital caliper. The calculations of the tumor volumes for spherical masses were done using (4/3 π)(r^3^) and for non-spherical masses (Length × Width × Height). All gross lesions and masses were then collected and fixed in 10% neutral buffer formalin (NBF). Lungs and any lesions were trimmed the same day and processed overnight. Tissues were embedded in paraffin, and sectioned at approximately 5 μm. Hematoxylin and eosin (H & E) stained slides were prepared each of the five separate lung lobes and from masses seen at necropsy. The tumor counts were based on histopathological analysis.

Slides were examined by a board certified veterinary pathologist using light microscopy or polarized light, which was occasionally used to confirm the presence or absence of foreign material (presumptive test material). The severity of non-neoplastic lesions was graded on a 4-point scale of minimal (1), mild (2), moderate (3), or marked (4) using an adaptation of previously described methods [104]. Presumptive MWCNT (foreign material) was recorded when present without severity grade [104]. Focal adenomatous alveolar hyperplasia was characterized by increased numbers of crowded alveolar epithelial cells that outlined contiguous alveolar septa in discrete, generally random locations (Figure 3). Severity was considered minimal, mild, moderate, or marked if roughly <5, 5–10, 11–20, or >20 contiguous alveoli were affected, respectively. Severity was increased or decreased a grade based on cell density and crowding. Histologic diagnoses were entered into the Provantis® data collection and management system. All lung slides from 10% of the mice in the terminal sacrifice were randomly selected for evaluation by a second board-certified veterinary pathologist who independently evaluated the slides while blinded to the interpretation of the study pathologist. There was 100% concordance on the diagnosis of neoplasia and 87% concordance on the diagnosis of adenocarcinoma versus adenoma. The differences were in the diagnosis of adenocarcinoma versus adenoma were considered by both the peer review and study pathologist to involve borderline lesions where such differences would be expected.

Tumor multiplicity (Table 2) was analyzed two ways. First, the number of tumors was divided by the number of animals that had tumors in each treatment group and was labeled “mean # of lung tumor per mice with tumors”. Secondly, the multiplicity was determined by dividing the number of tumors by the number of animals in the treatment group and was labeled “lung multiplicity adjusted by the total number of mice” (Table 2).

### Immunofluorescent detection of markers for mesothelioma

Immunofluoresecent staining for mesothelial proteins were performed on sarcomatous tumors of the peritoneal and epididymal surface in mice exposed to both MWCNT and MCA. Proteins identified by immunofluorescence were cytokeratins (wide spectrum), vimentin and podoplanin based upon their previously described expression in mouse or human mesothelioma [34,105-107]. To localize sites of vimentin and podoplanin expression to sites of cytokeratin expression, the immunofluorescence staining used double labeling for: 1) cytokeratin and vimentin, and 2) cytokeratin and podoplanin. Immunofluorescence was selected because it is more sensitive for identifying fluorescence in long, thin cytoplasm such as in alveolar type I cells or normal mesothelial cells [108].

For immunofluorescent staining, slides were deparaffinized, antigenicity was retrieved using EDTA, and non-specific reactivity was blocked with normal donkey serum (017-000-121, Jackson ImmunoResearch Laboratories, West Grove, PA), as previously described [108]. Two primaries from different species were used for each double label. Primary antibodies were hamster anti-podoplanin (NB600-1015, Novus Biologicals, Littleton, CO), mouse anti-pancytokeratin (C2652, Sigma-Aldrich, St Louis, MO), rabbit anti-vimentin (GTX62264, GeneTex, Irvine, CA), and rabbit anti-wide spectrum cytokeratin (rabbit ab9377). Secondary anti-bodies were DyLight 488, donkey anti-mouse (715-486-150, lot# 97733, Jackson ImmunoResearch Laboratories, West Grove, PA), DyLight 594 goat anti-hamster (107-515-142, lot 90054, Jackson ImmunoResearch Laboratories, West Grove, PA), Dylight 488 F(ab’)2 donkey anti-rabbit and DyLight 594 donkey anti-rabbit (711-516-152, lot 97356, Jackson ImmunoResearch Laboratories, West Grove, PA). Nuclei were stained using DAPI Fluoro Pure and slides were cover slipped with Prolong anti-fade reagent. Negative control slides were treated identically except that the primary antibody was replaced with non-immune serum from the same species as the primary antibody. Positive control slides were the normal mesothelial lining of the liver and lung.

### Enhanced-darkfield light microscopy imaging of nanoparticles

Carbon nanotubes in sections from exposed lungs were assessed using an enhanced-darkfield optical system as previously described [6]. Nanomaterials, such as carbon nanotubes, have dimensions less than the wavelength of light, have closely packed atoms, and typically have a refractive index significantly different from that of biologic tissues and/or mounting medium. These characteristics produce significantly greater scattering of light by nanoparticles than by the surrounding tissues and are visible with high contrast when examined with an enhanced-darkfield optical system designed to image scattered light in the section.

The optical system consisted of high signal-to-noise, darkfield-based illumination optics adapted to an Olympus BX-41 microscope (CytoViva, Auburn, AL 36830). Sections for dark-field examination were cut from paraffin blocks and collected on ultrasonically cleaned, laser cut slides (Schott North America Inc., Elmsford, N.Y. 10523) to avoid nanoparticle contamination from the ground edges of traditional slides. After staining with hematoxylin and eosin, sections were coverslipped with Permount. After alignment of the substage oil immersion optics with a 10× objective, sections were examined with 60× air or 100× oil immersion objectives. Enhanced darkfield images were taken with a 2048 × 2048 pixel digital camera (Dage-MTI Excel digital camera XLMCAT, Michigan City, In 46360).

### Statistical analysis

All analyses were performed using SAS/STAT version 9.3 for Windows. Binary outcomes of tumor incidence (tumor or not) for each type and for the total were analyzed using Fishers Exact test. Tumor counts were analyzed using Poisson regression for total tumor counts. In cases where Poisson regression demonstrated overdispersion a negative binomial regression was used. All analyses were stratified by promoter. Using the Proc Lifetest procedure in SAS, the log-rank test gave a p-value of 0.1609 for any differences among the 4 treatment groups with respect to the survival curves. Stratifying by initiator, the p-value with respect to the difference between MWCNT and Air was 0.3055, and 0.1288 for the corn oil and MC treated animals respectively.

## Competing interests

The authors declare that they have no competing interests.

## Authors’ contributions

LS conceived of the study and designed the study, analyzed the experimental results and drafted the manuscript. DTL contributed to the study design, conducted the animal experiments as well as contributed to the analysis of the data. AFH performed the gross pathology observations, pathology analysis, and was involved in the writing of the manuscript. LMS performed pathology analysis. KJS assisted in conducting the animal experiments and data analysis and contributed to the writing of the manuscript. MLK contributed to study design and performed statistical analysis. RBM performed analysis of the nanotubes in tissue sections. AKB contributed to the study design and experimental protocols for initiation. BTC, WM and DF performed inhalation experiments and contributed to design of the experiments. BTC and JLS were involved in acquisition of funding and writing of the manuscript. BTC was involved in the planning of the experiments. LB performed the necropsy, gross tumor counts and conducted lung preparation for histopathology. MA performed data analysis. ME and ST conducted the materials science characterization, contributed to study design and manuscript writing. KLF performed immunohistochemical analysis. DWP contributed a critical selection of the dose of the carbon nanotubes. SHR, VC and DWP contributed to the experimental design, acquisition of funding and writing of the manuscript. All authors read and approved the final manuscript.

## Supplementary Material

Additional file 1: Table S1 The table summarizes the mean exposure concentration of MWCNT material in the inhalation chamber for each of the animal exposure periods. The data is expressed in milligrams of MWCNT per meter cubed as well as the total MWCNT concentration for 5hours per day for a total of 15days. The measurements of the MWCNT material was based on data collected from Data RAM and filter samples. Click here for file
