# Supplementary material for: Promotion of lung adenocarcinoma following inhalation exposure to multi-walled carbon nanotubes
Source: Part Fibre Toxicol. 2014 Jan 9;11:3. doi: 10.1186/1743-8977-11-3 (PMC3895742; doi:10.1186/1743-8977-11-3)
Supplement: Additional file 1: Table S1 — The table summarizes the mean exposure concentration of MWCNT material in the inhalation chamber for each of the animal exposure periods. The data is expressed in milligrams of MWCNT per meter cubed as well as the total MWCNT concentration for 5hours per day for a total of 15days. The measurements of the MWCNT material was based on data collected from Data RAM and filter samples. [file 1743-8977-11-3-S1.doc]

| **Supplementary table 1: Summary of MWCNT exposures in the MWCNT/Cancer Study** | | | | | |
| --- | --- | --- | --- | --- | --- |
| Daily Exposure Concentration and Duration | | | | | Accumulated Exposure Dose |
| Exp # | Mean Conc. mg/m^3^ | Variation, % | Mean Exp. Time/day, min | Variation, % | Conc x Time, mg/m^3^ x hr |
| 1 | 4.6 | 6.6 | 327 | 2.5 | 372 |
| 2 | 4.7 | 7.4 | 323 | 1.5 | 379 |
| 3 | 4.6 | 4.5 | 326 | 1.7 | 375 |
| 4 | 4.6 | 5.4 | 328 | 2.7 | 376 |
| 5 | 4.6 | 7.5 | 323 | 1.6 | 373 |

**Supplementary table 1:**

The table summarizes the mean exposure concentration of MWCNT material in the inhalation chamber for each of the animal exposure periods. The data is expressed in milligrams of MWCNT per meter cubed as well as the total MWCNT concentration for 5 hours per day for a total of 15 days. The measurements of the MWCNT material was based on data collected from Data RAM and filter samples.
